# The complete mitochondrial genome of *Costapex baldwinae* (Gastropoda: Neogastropoda: Turbinelloidea: Costellariidae) from the Caribbean Deep-Sea

**DOI:** 10.1080/23802359.2021.1889408

**Published:** 2021-03-16

**Authors:** Juan E. Uribe, Alexander E. Fedosov, Katherine R. Murphy, Makiri Sei, Myroslaw G. Harasewych

**Affiliations:** a Department of Biodiversity and Evolutionary Biology, Museo Nacional de Ciencias Naturales (MNCN-CSIC), Madrid, Spain; b A.N. Severtsov Institute of Ecology and Evolution Russian Academy of Sciences, Moscow, Russia; c Laboratories of Analytical Biology, National Museum of Natural History, Smithsonian Institution, Washington, DC, USA; d Department of Invertebrate Zoology, National Museum of Natural History, Smithsonian Institution, Washington, DC, USA

**Keywords:** Mitogenome, Caribbean Sea, Bathyal depth, sunken wood, phylogenetics

## Abstract

We report the complete mitochondrial genome sequence of *Costapex baldwinae*, a Caribbean representative of a predominantly Indo-Pacific genus of gastropods that occurs on sunken wood at bathyal depths. The mitogenome is 15,321 bp in length and has a base composition of 29.2% A, 41.8% T, 12.0% C and 17.0% G. It contains 13 protein-coding, two ribosomal RNA, and 22 tRNA genes with the same gene order and strand orientation as other non-toxoglossan neogastropods. Phylogenetic analyses indicate that the superfamily Turbinelloidea, represented by this species, diverged early within the Neogastropod radiation, forming the sister group to a clade that includes five of the seven presently recognized superfamilies.

The Order Neogastropoda comprises an extremely diverse group of carnivorous marine gastropods with origins in the Early Cretaceous (Vermeij 1977; Tracey et al. 1993). Extensive initial diversification produced few diagnostic synapomorphies in the rapidly anastomosing clades (Harasewych et al. 1997). Investigations into phylogenetic relationships among the numerous lineages are ongoing and have resulted in a proliferation of family-level taxa, currently apportioned among seven superfamilies [Buccinoidea (9 families), Conoidea (17 families), Mitroidea (3 families), Muricoidea (1 family), Olivoidea (5 families), Turbinelloidea (5 families), Volutoidea (6 families)] as well as three families currently not assigned to superfamilies - Babyloniidae, Harpidae, Stepsiduridae (WoRMS 2020). Phylogenetic hypotheses of relationships among these superfamilies and families remain dynamic, with tree topologies varying depending on taxon sampling and types of data (e.g., Cunha, et al. 2009; Osca et al. 2015; Fedosov et al. 2015; Abdelkrim et al. 2018; Harasewych et al. 2020). We report the complete mitogenome of *Costapex baldwinae*, the first representative of the family Costellariidae and the superfamily Turbinelloidea for which such data are available, and evaluate its phylogenetic position within the Neogastropoda.

Genomic DNA (gDNA) was extracted from ∼20 µg of tissue sampled from a specimen of *Costapex baldwinae* Harasewych, Uribe & Fedosov, 2020 [United States National Museum, USNM 1622211, https://collections.nmnh.si.edu/search/iz/?irn=15881310, William Moser, moserw@si.edu] taken from a palmetto frond collected in 309 m off the Sea Aquarium, Bapor Kibra, Willemstad, Curaçao, 12°04.90'N, 63°53.74'W, preserved in absolute ethanol and stored at −20 °C. DNA was extracted using the Qiagen DNeasy Blood & Tissue kit following manufacturer’s spin-column protocol. A portion of the 16S rRNA gene was PCR-amplified and Sanger-sequenced using the primers and protocols in Uribe et al. (2020) and served as the initial reference sequence for assembly of the mitogenome. The mitogenome was amplified in two fragments with an overlap within the 16S rDNA gene that were produced using long PCR (protocol in Uribe et al. 2017) with the primer pairs: Cdeacox3F (Uribe et al., 2017) (5′–ATGGCACGAAATCCAATTTCATTTRGTTGA-3′) with 007-16S-R (5′-GAAGCTTTATTTGTTCCTCAGTCGC-3′) and 007-16S-F (5′-GGCTAGTATGAAGGGTTTGACAAG-3′) with CdeaPheR (Uribe et al., 2017) (5′-TACYTTAGCATCTTCAGCGCTAYGCTCT-3′). The two amplicons were then pooled for NGS library construction and sequenced on an Illumina MiSeq (Illumina, San Diego, CA) at the Laboratories of Analytical Biology of the NMNH following protocols in Uribe et al. (2020), resulting in 45,985 reads with an average length of 241 bp (±27.6).

The mitogenome was assembled following the protocol in Harasewych et al. (2020), using the partial 16S rRNA sequence as initial reference. A total of 44,053 reads mapped to the mitochondrial genome. Coverage ranged from 0 to 1833 per site (692.7 ± 676.1). The size and sequence of the region between the NAD5 and COX3 genes were determined using standard PCR and Sanger sequencing techniques, with primers derived from flanking regions: CB-nd5F (5′-GCCAAGGAACTAATGCCGTTCTTAG-3′) and CB-cox3R (5′-AAAGTGGCTTCTCGAATAACGTCCC-3′) in order to fill a small (136 bp) gap in the assembled mitogenome. Mitochondrial elements were annotated using MITOS (Bernt et al. 2013), ARWEN 1.2 (Laslett & Canbäck 2008) and the ORF finder in Geneious®.

The mitogenome of *Costapex baldwinae* (GenBank Acc. no. MW044625) is a double-stranded circular molecule 15,321 bp in length, and is composed of 29.2% A, 41.8% T, 12.0% C and 17.0% G. It contains 13 protein-coding, two ribosomal RNA, and 22 tRNA genes, all coded on the heavy strand (+) except for the tRNA cluster MYCWQGE and tRNA-T, which are coded on the light strand. Protein-coding genes span 11,246 bp (73.4%), rRNA genes 2324 bp (15.2%), tRNA genes 1509 bp (9.8%) of the mitogenome. Twenty-three intergenic regions 272 bp in total (1.8% of the mitogenome), range in size from 1 to 63 bp, the largest between COX1 and COX2, while the second largest between tRNA-F and COX3, presumably includes the origin of replication (Cunha et al. 2009). There are six overlapping regions, ranging in size from 1–16 bp (30 bp total), the largest (16 bp) between the NAD1 and tRNA-P genes.

Phylogenetic analyses indicate that Costellariidae, represented by *Costapex baldwinae*, is part of a lineage that diverged early in Neogastropod evolution, subsequent to Volutoidea, but prior to Olivoidea, Muricoidea, Buccinoidea, Mitroidea and Conoidea (Figure 1). This lineage has been provisionally ascribed to the superfamily Turbinelloidea, which presently includes the highly divergent families Volutomitridae, Ptychatractidae, Columbariidae and Vasidae (Fedosov et al. 2015), all with uncertain relationships to Turbinellidae. The monophyly of Turbinelloidea remains to be confirmed.

**Figure 1.**
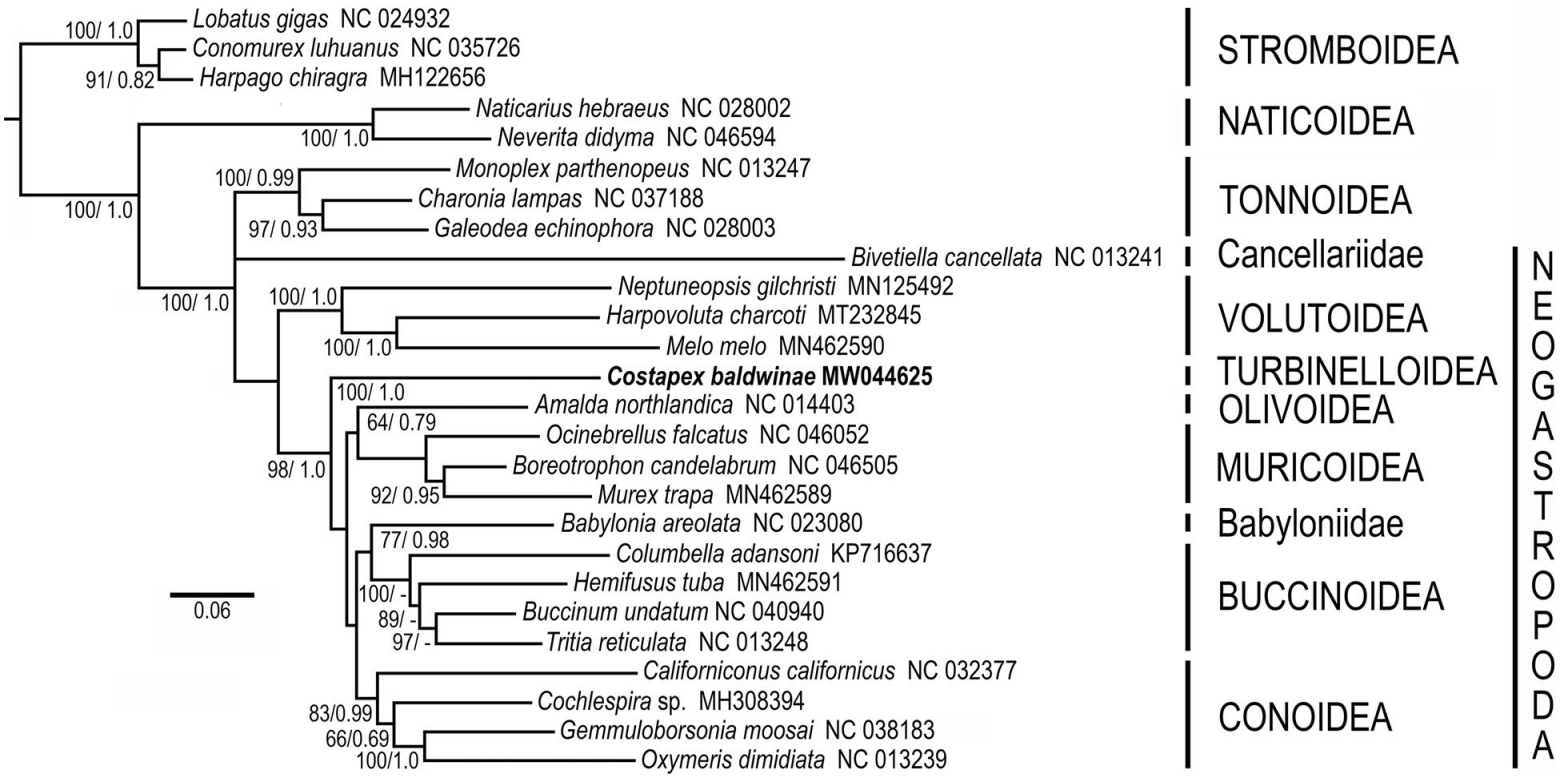
Relationships of *Costapex baldwinae* to other members of the Neogastropoda. Amino acid sequences of all protein-coding genes were individually aligned using MAFFT v. 7 (Katoh et al. 2019), their ambiguous positions removed using BMGE (Criscuolo and Gribaldo, 2010), then concatenated, producing an alignment with 3647 amino acid positions. Maximum likelihood analyses (1,000 independent tree searches and ultrafast-bootstrap runs) were performed using IQ-TREE (Nguyen et al. 2014) employing the best-fit mixture model selected by ModelFinder (Kalyaanamoorthy et al. 2017) with Bayesian Information Criterion (BIC) and the option -m TESTONLYNEW (mtMet+F+C50+R5). Phylogenetic analyses were run using an unpartitioned matrix. Bayesian inference analysis was performed using PhyloBayes MPI v.1.5a (Lartillot et al. 2013) running two independent MCMC chains until convergence under a CAT-GTR model and ‘-dc’ option with sampling every cycle. All runs consisted of >20,000 cycles and consensus trees were obtained after discarding the first 10% cycles as burn-in. Support shown as Bayesian posterior probability (when > 0.5).

## Data Availability

The mitogenome sequence data that support the findings of this study are openly available in GenBank at (https://www.ncbi.nlm.nih.gov/) under the accession number MW044625. The associated Bioproject, SRA and Biosample numbers are PRJNA698770, SRP304231, and SAMN17736058 respectively.
